# An Efficient Method for Magnetic Field Extrapolation Based on a Family of Analytical Three-Dimensional Magnetohydrostatic Equilibria

**DOI:** 10.1007/s11207-025-02469-1

**Published:** 2025-05-14

**Authors:** Lilli Nadol, Thomas Neukirch

**Affiliations:** School of Mathematics and Statistics, University of St Andrews, St Andrews, KY16 9SS UK

**Keywords:** Magnetic fields, models, Magnetic fields, corona, Magnetic fields, chromosphere, Magnetic fields, photosphere, Magnetic fields, extrapolation

## Abstract

With current observational methods it is not possible to directly measure the magnetic field in the solar corona with sufficient accuracy. Therefore, coronal magnetic field models have to rely on extrapolation methods using photospheric magnetograms as boundary conditions. In recent years, due to the increased resolution of observations and the need to resolve non-force-free lower regions of the solar atmosphere, there have been increased efforts to use magnetohydrostatic (MHS) field models instead of force-free extrapolation methods. Although numerical methods to calculate MHS solutions can deal with non-linear problems and hence provide more accurate models, analytical three-dimensional MHS equilibria can also be used as a numerically relatively “cheap” complementary method. In this paper, we present an extrapolation method based on a family of analytical MHS equilibria that allows for a transition from a non-force-free region to a force-free region. We demonstrate how asymptotic forms of the solutions can help to increase the numerical efficiency of the method. Through both artificial boundary condition testing and a first application to observational data, we validate the method’s effectiveness and practical utility.

## Introduction

Magnetic field extrapolation techniques are important tools for solar physics. This is due to the fact that, despite some progress (e.g. Landi et al. 2020), it is still not possible to obtain direct measurements of the coronal magnetic field routinely with sufficient resolution and accuracy. Instead, extrapolation uses magnetohydrodynamic (MHD) equilibrium solutions together with boundary conditions provided by photospheric magnetogram data to determine the magnetic field in the solar corona (e.g. Wiegelmann, Petrie, and Riley 2017; Wiegelmann and Sakurai 2021).

Because the (lower) corona usually has plasma beta ($\beta _{P}$) values that are (significantly) below unity, traditionally extrapolation methods have been based on force-free magnetic fields (e.g Wiegelmann, Petrie, and Riley 2017; Wiegelmann and Sakurai 2021). However, with the resolution of the data available now it might be necessary for extrapolation methods to take into account the lower layers of the solar atmosphere, which are not force-free (e.g. Metcalf et al. 1995). Therefore, in recent years magnetohydrostatic (MHS) extrapolation methods have been used as an alternative to force-free models, because they can accommodate for a non-force-free photosphere and chromosphere (for an overview, see Zhu, Neukirch, and Wiegelmann 2022).

The MHS equations are given by

$$\begin{aligned} \textbf{j} \times \textbf{B} - \nabla p - \rho \nabla \Psi &= 0, \\ \nabla \times \textbf{B} &= \mu _{0} \textbf{j}, \\ \nabla \cdot \textbf{B} &= 0, \end{aligned}$$

where $\mathbf{B}$ denotes the magnetic field, $\mathbf{j}$ the current density, $p$ the plasma pressure, $\rho $ the mass density, $\Psi $ the gravitational potential, and $\mu _{0}$ the permeability of free space. In this paper, we will use a Cartesian coordinate system with $z$ being the height above the solar surface. With a constant gravitational acceleration, $g$, acting in the negative $z$-direction, the gravitational potential takes the form $\Psi =gz$.

In general, these equations are non-linear and therefore numerical methods are required to solve them for extrapolation purposes (e.g. Gilchrist and Wheatland 2013; Zhu and Wiegelmann 2018), which is computationally expensive. In this paper we shall discuss the alternative possibility of using analytical 3D MHS solutions for extrapolation purposes. To be able to solve the 3D MHS equations analytically one has to make a number of simplifying assumptions, which lead to a linear mathematical problem not only allowing for analytical solutions but also for the superposition of different solutions. This usually allows for a quicker and more efficient way of MHS extrapolation, but the assumptions made to simplify the mathematical problem may also limit the applicability of extrapolation methods based on analytical MHS solutions. Such methods should therefore not be viewed as a replacement of numerical methods based on the full non-linear MHS problem, but as complementary to those methods.

The general method we use to calculate analytical 3D MHS solutions is based on a series of papers by Low (1985, 1991, 1992) (see also Bogdan and Low 1986; Low 1993a,b, 2005). Magnetohydrostatic equilibria determined by applying this general method in Cartesian, cylindrical and spherical coordinate systems have been used by many authors (e.g. Bagenal and Gibson 1991; Gibson and Bagenal 1995; Gibson, Bagenal, and Low 1996; Neukirch 1995, 1997b, 2009; Aulanier et al. 1998, 1999; Zhao and Hoeksema 1993, 1994; Zhao, Hoeksema, and Scherrer 2000; Petrie 2000; Ruan et al. 2008; Al-Salti, Neukirch, and Ryan 2010; Al-Salti and Neukirch 2010; Gent et al. 2013; MacTaggart et al. 2016; Wilson and Neukirch 2018).

Low (1991, 1992) introduced a specific class of analytical MHS magnetic fields for which the non-force-free terms decay exponentially with increasing height. Such a transition generally agrees with the change of the plasma beta, $\beta _{P}$, in the solar atmosphere (e.g. Gary 2001). These solutions have been used for magnetic field extrapolation using Sunrise/IMaX data as boundary conditions (Wiegelmann et al. 2015, 2017).

More recently, Neukirch and Wiegelmann (2019) have proposed a new family of MHS solutions showing a transition from a non-force-free to a force-free domain. This transition is based on a hyperbolic tangent dependence on height $z$ rather than an exponential dependence as in Low (1991, 1992). The family of solutions by Neukirch and Wiegelmann (2019) allows more control over the properties of the transition. However, this increase in control is gained at the expense of an increase in model parameters. In particular, the Neukirch and Wiegelmann (2019) solutions allow for the height at which the non-force-free to force-free transition occurs and the width over which it takes place to be specified. Hence, it is possible to fit the transition in the nature of the magnetic field to the height of the transition region between the chromosphere and the solar corona.

So far the MHS solutions by Neukirch and Wiegelmann (2019) have only been applied to a simple bipolar “toy” magnetogram and not to real data. The main reason for this is that the analytical solution involves hypergeometric functions and these can be difficult to evaluate numerically for certain parameter combinations. The main aim of this paper is to resolve these problems and to apply the new solution family to observational data for the first time. To achieve this aim, we have found a method to approximate the hypergeometric functions by a piecewise continuously differentiable combination of exponential functions, which represent an asymptotic solution of the mathematical problem for small widths of the region in which the transition from non-force-free to force-free takes place. Before applying the asymptotic solution to observational data we present the results of a controlled test model showing that the asymptotic solution substantially increases the numerical efficiency without compromising the accuracy of the solutions. It is therefore a good basis for generating an efficient numerical extrapolation tool based on the family of MHS solutions by Neukirch and Wiegelmann (2019).

The structure of the paper is as follows. In Section 2 we summarise the basic theory of the particular class of 3D MHS solutions by Neukirch and Wiegelmann (2019). In Section 3 we present the new asymptotic solution and how we test it, the datasets used as examples, and the analysis tools used to quantitatively assess the quality of the approximation of the exact solutions by the asymptotic solution. Section 4 describes the results of our investigations, followed by the final discussion in Section 5.

## Background Theory

As stated before, we use Cartesian coordinates with $z$ being the height above the solar surface. We follow the approach proposed by Neukirch and Rastätter (1999) and use a poloidal–toroidal decomposition (e.g. Chandrasekhar and Kendall 1957; Nakagawa and Raadu 1972) for representing the magnetic field,

1 $$ \textbf{B} = \nabla \times \left [ \nabla \times \left ( \Phi \hat{\textbf{z}} \right ) \right ] + \nabla \times \left ( \alpha \Phi \hat{\textbf{z}} \right ). $$

Here, $\alpha $ is assumed to be constant, which results in the toroidal function $\Theta $ to take the form $\Theta = \alpha \Phi $ (e.g. Nakagawa and Raadu 1972).

Following Low (1991) we assume a current density of the form

2 $$ \mu _{0} \textbf{j} = \alpha \textbf{B} + \nabla \times \left (f(z)B_{z} \hat{\textbf{z}}\right ) = \alpha \textbf{B} + f(z) \nabla B_{z} \times \hat{\textbf{z}}, $$

such that the non-force-free contribution to the current density in Equation 2 evolves according to $f(z)B_{z}$ with height $z$. Therefore, if $f(z)$ tends to zero for $z \to \infty $ the perpendicular part of the current density also tends to zero, which leads to the magnetic field approaching a (linear) force-free state.

The poloidal function $\Phi $ defining the magnetic field in Equation 1 is determined by solving Ampère’s law in the form (Neukirch and Rastätter 1999)

3 $$ \Delta \Phi - f(z) \Delta _{xy} \Phi + \alpha ^{2} \Phi = 0 , $$

where $\Delta _{xy} \Phi = \frac{\partial ^{2} \Phi}{\partial x^{2}} + \frac{\partial ^{2} \Phi}{\partial y^{2}}$. Once $\Phi $ is known, the magnetic field can be found via Equation 1).

One can integrate the force balance equation (see e.g. Low 1991; Neukirch and Rastätter 1999) and obtain expressions for the plasma pressure and mass density in the form

4 $$\begin{aligned} p(x,y,z) =& p_{b}(z) + \Delta p(x,y,z) = p_{b}(z)-f(z) \frac{B_{z}^{2}}{2 \mu _{0}}, \end{aligned}$$

5 $$\begin{aligned} \rho (x,y,z) =& -\frac{1}{g}\frac{d p_{b}}{dz} + \Delta \rho (x,y,z)= \frac{1}{g} \left ( - \frac{d p_{b}}{dz} + \frac{df}{dz} \frac{B_{z}^{2}}{2 \mu _{0}} + \frac{f}{\mu _{0}} \textbf{B} \cdot \nabla B_{z} \right ), \end{aligned}$$

where $p_{b}$ is a free function of height $z$, which arises during the integration of the force balance equation (an “integration constant”). This function can be interpreted as the plasma pressure of a stratified background atmosphere and while there are no mathematical restrictions on $p_{b}(z)$, from a physics point of view it has to satisfy two conditions: (i) it has to be strictly positive, i.e. $p_{b}(z) > 0$ for all $z \ge 0$ and (ii) it has to be a strictly monotonically decreasing function of $z$, i.e. $\frac{d p_{b}}{dz} < 0$ for all $z$. The reason for these two conditions is that this is the only way to get a positive total plasma pressure and density. The plasma temperature can then be obtained from $p$ and $\rho $ using the ideal gas law $T = \bar{\mu}p / (k_{B} \rho )$, where $\bar{\mu}$ is the mean atomic weight of the plasma and $k_{B}$ the Boltzmann constant.

A convenient way of solving the partial differential equation 3 for $\Phi $ exploits the fact that its coefficients only depend on $z$. Therefore one can use Fourier transforms in the $x$- and $y$-coordinates, i.e. write

6 $$ \Phi = \iint _{- \infty}^{\infty }\bar{\Phi}(z; k_{x}, k_{y}) \exp \left [ i \left (k_{x} x + k_{y} y \right ) \right ] dk_{x} dk_{y} . $$

The scalar function $\bar{\Phi}$ is the solution of the linear ordinary second-order differential equation

7 $$ \frac{d^{2} \bar{\Phi}}{dz^{2}} + \left [ \alpha ^{2} - k^{2} + k^{2} f(z) \right ] \bar{\Phi} = 0, $$

where $k^{2} = k_{x}^{2} + k_{y}^{2}$. If periodic boundary conditions in $x$ and $y$ are imposed $k_{x}$ and $k_{y}$ take on discrete values and the integrals in Equation 6 become sums.

For $f(z)=f_{0} =\text{constant}$, the solutions to Equation 7 are exponential functions (e.g. Neukirch 1997a; Petrie 2000; Petrie and Neukirch 2000). Low (1991) used an exponentially decaying $f(z)$, i.e.

8 $$ f_{L}(z) = a_{L} \exp \left (- \kappa z \right ) $$

with magnitude parameter $a_{L}$ and inverse length scale $\kappa $. For this $f(z)$, the solutions of Equation 7 for $\bar{\Phi}$ are Bessel functions. More recently, Neukirch and Wiegelmann (2019) suggested a more complicated $f(z)$ of the form

9 $$ f_{N+W}(z) = a \left [ 1 - b \tanh \left ( \frac{z - z_{0}}{\Delta z} \right ) \right ]. $$

The corresponding solutions for $\bar{\Phi}$ can be expressed in terms of hypergeometric functions.

**Figure 1:**
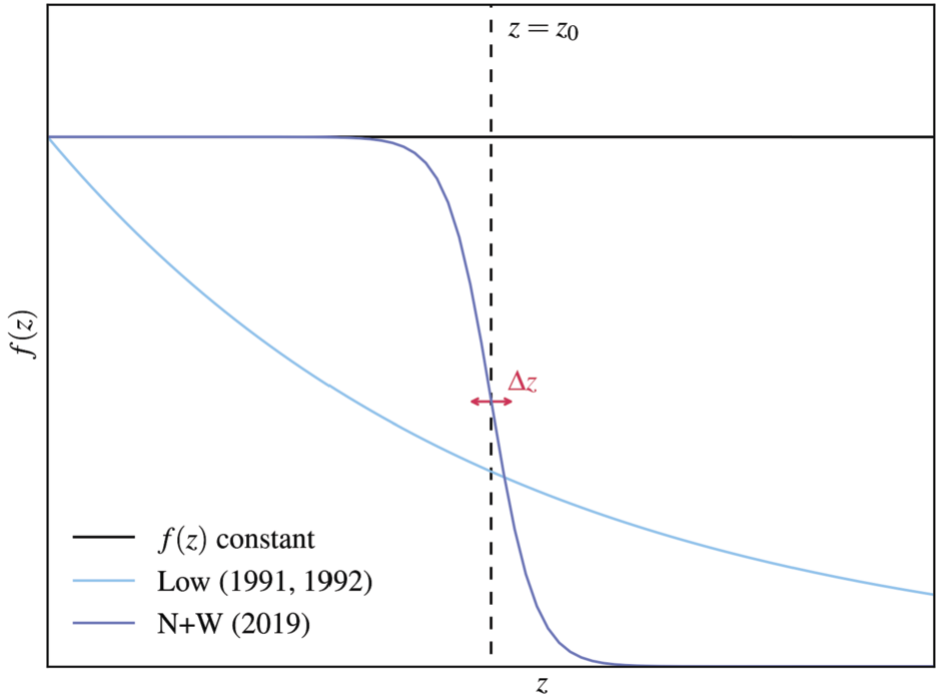
Different choices for $f(z)$. The black line corresponds to $f(z)=$ constant, the light blue curve to the exponential function used by Low (1991), and the dark blue curve to the hyperbolic tangent profile from Neukirch and Wiegelmann (2019). This figure is representative of the case where $b=1.0$ in Equation 9.

These three different choices for the function $f(z)$ are shown in Figure 1 for a specific combination of parameters in Equation 9 ($b=1.0$, $a_{L} = a (1- \tanh (-z_{0} / \Delta z)$, $\kappa = 1/z_{0}$, and the constant $f(z)$ such that it matches $f_{L}$ and $f_{N+W}$ on the photosphere). As one can see in Figure 1, if the argument of the hyperbolic tangent function in $f_{N+W}(z)$ is large and negative the function is close to the asymptotic value $a(1+b)$, whereas in tends to the value $a(1-b)$ if the argument of the hyperbolic tangent is large and positive. Therefore, the parameter $a$ controls the overall magnitude of $f_{N+W}$, whereas $b$ controls the difference between the two asymptotic values of $f_{N+W}(z)$. In the case $b=1$, the parameter $b$ can be considered the “switch off” parameter, because the asymptotic value of $f_{N+W}(z)$ for $(z-z_{0})/ \Delta z \gg 0$ is zero in this case, implying that the magnetic field tends towards a linear force free field in this limit. The example for $f_{N+W}(z)$ shown in Figure 1 is for $b=1$. As Figure 1 also shows, $z_{0}$ is the central height around which the transition from non-force-free to force-free magnetic field occurs and $\Delta z$ controls the width over which that transition takes place. If $b \neq 1$ a certain degree of non-force-freeness can be maintained in the upper part of the model above $z_{0}$ (Neukirch and Wiegelmann 2019). We remark that a similar effect could be introduced into $f_{L}(z)$ by adding a constant parameter $b_{L}$ to the exponential function. The $f_{N+W}(z)$ profile has more parameters than the $f_{L}(z)$ function. The aim is to have more control over the transition from the non-force-free to force-free domain and, as a consequence, more control over the resulting pressure and density profiles.

The solution of Equation 7 with Equation 9 by Neukirch and Wiegelmann (2019) is given by

10 $$ \bar{\Phi}_{N+W} = \bar{A} \eta ^{\bar{\delta}} \left (1-\eta \right )^{ \bar{\gamma}} {}_{2}F_{1} \left ( \bar{\gamma} + \bar{\delta} + 1, \bar{\gamma} + \bar{\delta}, 2 \bar{\delta} + 1; \eta \right ), $$

where ${}_{2}F_{1}(a, b, c; z)$ is the hypergeometric function.^1^ Here, the argument $\eta $ is defined as

11 $$ \eta = \frac{1}{2}\left [1-\tanh \left ( \frac{z-z_{0}}{\Delta z} \right ) \right ], $$

and the parameters are given by the expressions $\bar{\gamma} = \sqrt{C_{2}}$ and $\bar{\delta} = \sqrt{C_{1}}$, with

$$\begin{aligned} C_{1} &= \frac{1}{4} \left [ \bar{k}^{2} \left ( 1 - a + ab \right ) - \bar{\alpha}^{2} \right ], \\ C_{2} &= \frac{1}{4} \left [ \bar{k}^{2} \left ( 1 - a - ab \right ) - \bar{\alpha}^{2} \right ]. \end{aligned}$$

Here $\bar{k} = k \Delta z$ and $\bar{\alpha} = \alpha \Delta z$ (Neukirch and Wiegelmann 2019), and $\tilde{A}$ is determined by the boundary conditions. We remark that in Equation 10 we have only included the part of the general solution which tends to zero as $z \to \infty $ (for the full solution, see Neukirch and Wiegelmann 2019).

## Methodology

### Approximate Solutions in the Asymptotic Regime

The solution presented in Equation 10 for the case when $f_{N+W}$ is used in the ODE 7 is general, but it still presents some challenges due to the hypergeometric functions involved. Although hypergeometric functions are a standard class of special functions, it is not necessarily intuitively clear how their general behaviour changes depending on the parameter values used. Furthermore, the hypergeometric differential equation has singular points and it turns out that the solution 10 can approach one of these singular points. This can be problematic for the numerical evaluation of the hypergeometric functions with respect to both accuracy and speed even when using well-tested numerical implementations as, for example, the Python SciPy library. However, it turns out that one can use the properties of $f_{N+W}(z)$ and the resulting asymptotic behaviour of the ODE 7 in a certain parameter regime to obtain an approximate solution that can be expressed purely in terms of exponential functions. It is one of the main aims of this paper to present the derivation of this approximate solution in the asymptotic regime and to assess its accuracy in representing the exact solution. This, together with an assessment of the gain in computational speed, will allow us to evaluate whether and when this approximate solution can be safely used to replace the exact solution.

We define $\tilde{z} = z/L$, $\tilde{\alpha} = \alpha L$, $\tilde{k} = k L$, and $\Delta \tilde{z}= \Delta z/L$, with $L$ a general normalising length scale. The relation to the previous normalisation with $\Delta z$ as normalising length scale is simply given by $\bar{\alpha} = \tilde{\alpha} \Delta \tilde{z}$, $\bar{k} = \tilde{k} \Delta \tilde{z}$, and so on.

As already indicated in Section 2, the function $f_{N+W}(z)$ asymptotically tends to constant values for large negative or positive argument of the hyperbolic tangent function (i.e. for $|z-z_{0}| \gg \Delta z$ in Equation 9. If $\Delta \tilde{z}$ is small the transition between the asymptotically constant values of $f_{N+W}$ takes place over a very small domain in $\tilde{z}$, centred at $\tilde{z}_{0}$. In the domains where $f_{N+W}$ tends to its asymptotic values equal to $f \simeq a(1 \pm b)$, Equation 7 is reduced to

12 $$ \frac{d^{2} \bar{\Phi}}{d\tilde{z}^{2}} + \left [ \tilde{\alpha}^{2} - \tilde{k}^{2} \left ( 1 - a \pm ab \right ) \right ] \bar{\Phi} = 0, $$

with the + sign applying for $\eta = (\tilde{z} - \tilde{z}_{0})/\Delta \tilde{z} > 0$ and the − sign for $\eta < 0$. We define

$$ C_{\pm} =\frac{1}{4}\left [ \tilde{k}^{2} \left ( 1 - a \pm ab \right ) -\tilde{\alpha}^{2} \right ] $$

and assume that $C_{\pm}> 0$ (we note that $C_{1} = C_{+} \Delta \tilde{z}^{2} $ and $C_{2} = C_{-} \Delta \tilde{z}^{2} $). The above asymptotic limit is equivalent to using the alternative function

$$ f(z) = \textstyle\begin{cases} a (1-b) &\text{for } z < z_{0} \\ a (1+b) &\text{for } z > z_{0} \end{cases} $$

instead of Equation 9. This dicontinuous $f(z)$ introduces a jump in Equation 7, yielding Equation 12.

An approximation to the exact solution $\bar{\Phi}$ of Equation 7 can be found by solving Equation 12 in the two domains $0 \le \tilde{z} \le \tilde{z}_{0}$ and $\tilde{z}_{0} < \tilde{z} < \infty $, under the following conditions

i) $\bar{\Phi}$ is once continuously differentiable at $\tilde{z} = \tilde{z}_{0}$,
ii) $\bar{\Phi}\to 0$ as $\tilde{z} \to \infty $, and
iii) $\bar{\Phi}= 1$ at $z=0$.

Defining $\delta = \sqrt{C_{+}}$ and $\gamma = \sqrt{C_{-}}$, the solution is given by

13 $$ \bar{\Phi} = \frac{1}{D} \textstyle\begin{cases} \frac{\delta}{\gamma} \sinh \left [ 2 \gamma (\tilde{z}_{0}-\tilde{z}) \right ] + \cosh \left [ 2 \gamma (\tilde{z}_{0}-\tilde{z}) \right ] & \text{for } 0 \le \tilde{z} \le \tilde{z}_{0} \\ \exp \left [ - 2 \delta (\tilde{z}-\tilde{z}_{0}) \right ] & \text{for } \tilde{z}_{0} < \tilde{z} < \infty \end{cases}\displaystyle , $$

with

$$ D = \frac{\delta}{\gamma} \sinh \left ( 2 \gamma z_{0} \right ) + \cosh \left ( 2 \gamma z_{0} \right ). $$

In Equation 13, we have for ease of notation suppressed the dependence of $\bar{\Phi}$ on the value of $k^{2}$, linking it to the specific Fourier modes. We will use this simpler notation for the remainder of this paper.

The first derivative of $\bar{\Phi}$ is

14 $$ \bar{\Phi}' = - \frac{1}{D} \textstyle\begin{cases} 2 \delta \cosh \left [ 2 \gamma (\tilde{z}_{0}-\tilde{z}) \right ] + 2 \gamma \sinh \left [2 \gamma (\tilde{z}_{0}-\tilde{z})\right ] & \text{for } \le \tilde{z} \le \tilde{z}_{0} \\ 2 \delta \exp \left ( - 2 \delta (z-z_{0}) \right ) & \text{for } \tilde{z}_{0} < \tilde{z} < \infty \end{cases}\displaystyle . $$

**Figure 2:**
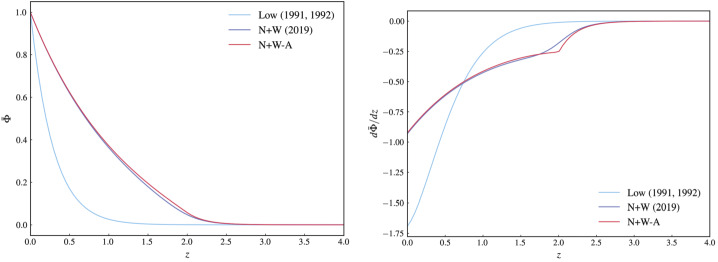
$\bar{\Phi}$ (left panel) and $d \bar{\Phi}/ d\tilde{z}$ (right panel) for a single Fourier mode ($\tilde{k}^{2} = 2\pi ^{2}\approx 19.7392$). Shown are the exact solution (labelled N+W (2019), dark blue), the approximate asymptotic solution (labelled N+W-A, red), and for comparison a solution based on $f_{L}(z)$ (labelled Low (1991, 1992), light blue). The parameter values used for the N+W and N+W-A curves are $\tilde{z}_{0} = 2.0$, $\Delta \tilde{z} = 0.2$, $a=0.48$, $b=1.0$ and $\tilde{\alpha} = 0.03$. The parameter values used for the Low (1991, 1992) curve are $\kappa = 1/\tilde{z}_{0}$ and $a_{L} = a (1+\tanh (\tilde{z}_{0} / \Delta \tilde{z}))$. All functions are shown over the domain $0 \le \tilde{z} \le 2\tilde{z}_{0}$ to include not only the region around $\tilde{z}_{0}$ where $f_{N+W}$ changes between its asymptotic values, but also parts of the regions outside this transitional domain. It is obvious from this plot that at least in this particular case the asymptotic solution and its first derivative are very good approximations of their exact counterparts. Unsurprisingly, the Low (1991, 1992) curve deviates significantly from both.

The behaviour of this approximate solution based on the asymptotic form of the ODE 12 is consistent with the asymptotic behaviour of the exact solution Equation 10, as found analytically by Neukirch and Wiegelmann (2019). An illustrative example for a typical combination of parameter values and a single value of $\tilde{k}^{2}$, i.e. a single Fourier mode, is shown in Figure 2. The figure displays plots of three different functions. In the left panel, we show the asymptotic solution derived above (labelled N+W-A), the exact solution (labelled N+W), and for comparison a solution based on $f_{L}(z)$ defined in Equation 8, using parameter values that are derived from the parameter values used for $f_{N+W}$ (for details we refer to the caption of Figure 2). The panel on the right shows plots of the first derivatives of these three functions. The asymptotic solution approximates the exact solution generally very well. As is to be expected, the largest deviations of the asymptotic solution from the exact solution can be seen in the region around $\tilde{z} \approx \tilde{z}_{0}$, and it is more pronounced in the derivative (right panel) than in the function itself. Not surprisingly, the Low (1991, 1992) solution differs significantly from the other two solutions, despite matching the parameter values for $f_{L}(z)$ and $f_{N+W}$ as much as possible.

Although this illustrative example is promising, an assessment of the quality of the approximation should not be based on a single Fourier mode and a specific parameter set. One of the major purposes of this paper is to present a detailed comparison between magnetic field extrapolations based on the exact solution and the asymptotic solution, and how the differences between the two depend on the parameters of $f_{N+W}$, in particular $a$ and $\Delta \tilde{z}$. This will allow a much more solid assessment of the quality of the approximation provided by the asymptotic solution. In this paper we will only investigate the case $b=1$, which means that the magnetic field very quickly approaches a linear force-free state above $\tilde{z}_{0}$.

The following considerations allow us to restrict the parameter range to sensible values. Firstly, we shall use the method by Seehafer (1978) to deal with unbalanced magnetograms. This implies that periodic boundary conditions are imposed in the $x$- and $y$-directions on the extended domain used by the Seehafer method. Hence, the solution of Equation 3 is given by a Fourier series instead of a Fourier transformation as in Equation 6. We define the wave number associated with the fundamental mode of this Fourier series as $\tilde{k}_{min}$. Following Neukirch and Wiegelmann (2019), we assume that $C_{-} > 0$ (this also implies $C_{2} > 0$) so that the parameter $\gamma $ (and $\bar{\gamma}$) only takes on real values. We note that due to $C_{+} \ge C_{-}$ ($C_{1} \ge C_{2}$) this also ensures that $\delta $ only takes on real values. As shown by Neukirch and Wiegelmann (2019), this condition means that the maximum possible value for $a$ is given by^2^

15 $$ a_{max} = \frac{1}{b+1}\left [1 - \left ( \frac{\tilde{\alpha}}{\tilde{k}_{min}}\right )^{2}\right ]. $$

We also impose $\tilde{\alpha}^{2} < \tilde{k}_{min}^{2}$ to keep $C_{+} > 0$ (for the case $b=1$).

The second important model parameter whose influence on the accuracy of the asymptotic solution has to be investigated is $\Delta \tilde{z}$. As already stated before, the idea behind the asymptotic solution is that $\Delta \tilde{z}$ is small, so that for most of the domain in $\tilde{z}$ the ODE 7 is well approximated by the asymptotic ODE 14. In other words, the value of $\Delta \tilde{z}$ has to be small enough so that the hyperbolic tangent function can be considered to be replaced by a step function in the ODE 7. We emphasise that this is only done to calculate $\bar{\Phi}_{N+W-A}$ and the resulting magnetic field, but that the exact form of $f_{N+W}(z)$ is used in the calculation of the pressure and the density. We discuss the reason for this below. The obvious question is which other length scale we compare $\Delta \tilde{z}$ with to decide whether it is small. Given that the argument of the hyperbolic tangent function in $f_{N+W}$ is $\eta = (\tilde{z} - \tilde{z}_{0})/\Delta \tilde{z}$, a natural choice for such a comparison is $\tilde{z_{0}}$. In this paper, we will therefore test the quality of the asymptotic solution only up to an upper bound of $\Delta \tilde{z} = \tilde{z}_{0}$. Because $\Delta \tilde{z}$ has to be small for the asymptotic solution to be accurate, it seems tempting to consider the limit $\Delta \tilde{z} \to 0$. In this case, the hyperbolic tangent would become a step function, and the asymptotic solution would actually become the exact solution. However, this is not a physically valid limit due to the expression for the density, given in Equation 5. This expression depends on the first derivative of $f$ with respect to $z$. In the limit $\Delta \tilde{z} \to 0$ this derivative turns into a Dirac $\delta $-function at $\tilde{z} = \tilde{z}_{0}$, leading to the density going to (negative) infinity at that point, which is clearly unacceptable. One therefore also needs to impose a finite lower bound on $\Delta \tilde{z}$ which is on the one hand small enough to make the asymptotic solution sufficiently accurate, but which on the other hand keeps the transition between the two asymptotic regions smooth enough. There is also another reason why the $\Delta \tilde{z}$ value cannot be chosen too small. It turns out that very small values for $\Delta \tilde{z}$ can cause numerical problems with the calculation of the exact solution. Hence we choose $\Delta \tilde{z}= 0.1 \tilde{z}_{0}$ as the typical value. We remark that in this case if one takes $L = 10^{6} $ m and $\Delta{z}_{0} = 2.0$ (i.e. $z_{0} = 2000$ km) one obtains a reasonable value for the width of the transitional region, namely $\Delta z = 200$ km.

### Boundary Conditions for Test Cases

To assess the quality of the approximation of the exact solution by the asymptotic solution, we consider two different photospheric boundary conditions for the magnetic field component $B_{z}$. As already mentioned before we use the method by Seehafer (1978) to deal with boundary data for which the magnetic flux through the lower boundary ($\tilde{z}=0$) is unbalanced. This implies periodic boundary conditions (on the extended domain required by the method) in the $x$- and $y$-directions.

The first boundary condition is given analytically and represents a generalisation to a multipole of the analytical periodic bipole boundary condition used by Neukirch and Wiegelmann (2019). The second boundary condition uses magnetogram data taken by the Solar Dynamics Observatory (SDO/HMI, Scherrer et al. 2012). This case tests whether the asymptotic method can be successfully applied to observational data, providing a more realistic assessment of the quality of the asymptotic solution method. Both cases also allow us to test the gain in numerical efficiency by using the much simpler asymptotic solution in comparison with the exact solution.

#### Analytical Multipole Boundary Conditions

The artificial boundary condition we use to test our method is constructed similarly to the bipole boundary condition in Neukirch and Wiegelmann (2019). The vertical magnetic field component at $z=0$ is given by

16 $$ B_{z}(x,y,0) = B_{0} \sum _{i=0}^{8} (-1)^{i+1} \frac{\exp (\lambda _{x,i} \cos (\hat{x}-\mu _{x,i}))}{2 \pi \text{I}_{0}(\lambda _{x,i})} \frac{\exp (\lambda _{y,i} \cos (\hat{y}-\mu _{y,i}))}{2 \pi \text{I}_{0}(\lambda _{y,i})}, $$

where $\text{I}_{0}$ is a modified Bessel functions of the first kind. To achieve a compact form of the argument of the cosine function in the exponentials in Equation 16 we have assumed that the region under consideration on the bottom boundary is $0\le x \le x_{0}$, $0\le y \le y_{0}$. We then define $\hat{x} = \pi \left ( 2x/x_{0}- 1\right )$ and $\hat{y} = \pi \left (2y/y_{0}-1 \right )$, so that $-\pi \le \hat{x}$, $\hat{y} \le \pi $.

**Figure 3:**
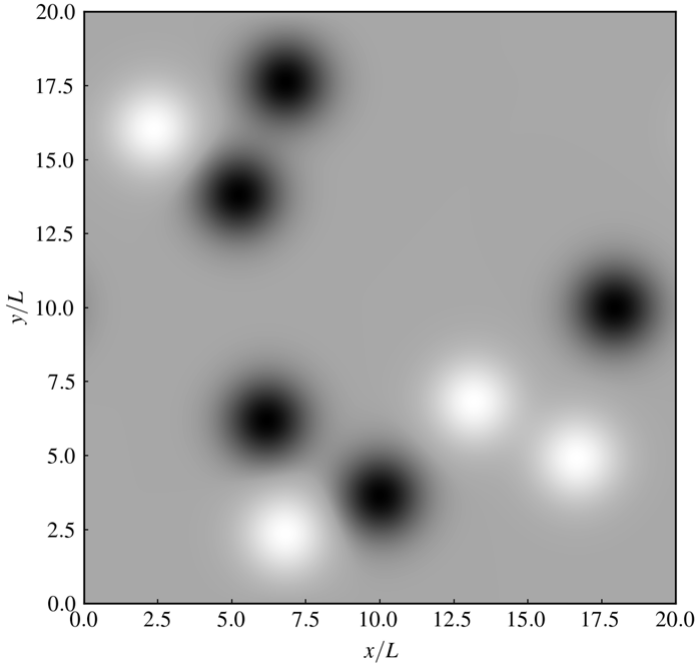
Artificial line-of-sight magnetogram created from Equation 16 using the parameters given in the text as well as in Table 1.

The terms in Equation 16 with odd $i$ correspond to regions of positive magnetic polarity and the ones with even $i$ correspond to regions of negative magnetic polarity. Because the sum has nine terms, the resulting magnetogram is not flux-balanced. We made this choice to have a controlled test of our implementation of the Seehafer (1978) method. The dimensionless parameters $\lambda _{x,i}$ and $\lambda _{y,i}$ can be regarded as normalised inverse length scales, which determine the width of each magnetic flux region in the $x$- and $y$-direction. We have chosen a uniform value of $\lambda _{x,i} = \lambda _{y,i} = \lambda = 10.0 $. The parameters $\mu _{x_{i}}$ and $\mu _{y_{i}}$ determine the positions of the centres of the magnetic flux sources within the computational domain. The specific values of all model parameters chosen for this case are listed in Table 1. The resulting photospheric $B_{z}$ is shown in Figure 3. In normalised coordinates, the computational domain has an extent of 20.0 in each direction. We choose $z_{0} = 2.0$, which for a normalising length scale $L=1$ Mm corresponds to a height of 2.0 Mm, which approximately corresponds to the height of the transition region in the solar atmosphere.

**Table 1:** Parameter values for the analytical multipole boundary condition case, Equation 16.

*i*	$\mu _{x,i}$	$\mu _{y,i}$	$\lambda _{x,i}$	$\lambda _{y,i}$
0	1.0	− 1.0	10.0	10.0
1	− 1.2	− 1.2	10.0	10.0
2	− 2.4	1.9	10.0	10.0
3	2.1	− 1.6	10.0	10.0
4	− 1.5	1.2	10.0	10.0
5	2.5	0.0	10.0	10.0
6	0.0	− 2.0	10.0	10.0
7	− 1.0	− 2.4	10.0	10.0
8	− 1.0	2.4	10.0	10.0

#### Observational Data Boundary Conditions

**Figure 4:**
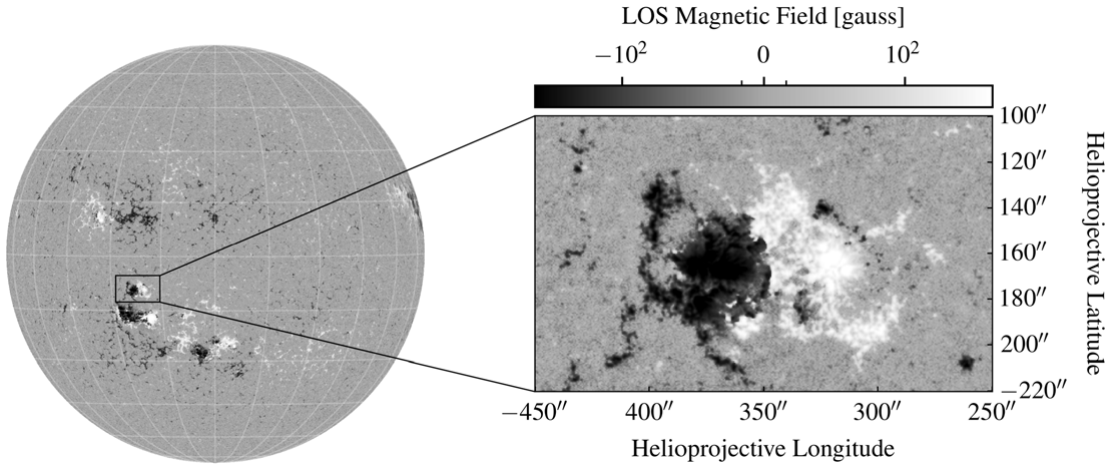
Full disk line-of-sight magnetogram observed by HMI onboard SDO on 2012 June 13 at 07:31:30 UT with zoomed in cutout of the active region used for the application of the extrapolation method based on the exact solution family by Neukirch and Wiegelmann (2019) and its asymptotic approximation.

In this paper we aim to apply the Neukirch and Wiegelmann (2019) MHS solutions family and the asymptotic approximation derived above to observational data for the first time. For this, we use a photospheric line-of-sight magnetogram taken by the Helioseismic and Magnetic Imager (HMI, Schou et al. 2012) onboard SDO. We choose an observation from 2012 June 13 at 07:31:30 UT as an example case for this study. The full disk line-of sight HMI magnetogram is shown in Figure 4 together with the zoomed-in cutout ($397 \times 239$ pixel) we use as boundary condition for magnetic field extrapolation. As one can see the cutout shows a largely bipolar magnetic polarity structure with some smaller substructure visible as well. This magnetogram was chosen because it will provide a very good test for our MHS extrapolation method in general, and for a comparison of the exact and asymptotic solution cases.

As the active region under investigation here is close to the centre of the disk no distinction has been made between $B_{LOS}$ and the radial field component $B_{r}$ in the heliographic projection, which should theoretically be used. Therefore, for the purpose of this paper, which is to compare the exact to the analytical solution, the differences between $B_{LOS}$ and $B_{r}$ are ignored.

### Analysis Tools

Because the MHS solution for the magnetic field $\boldsymbol{B}$ is given analytically, it can in principle be calculated at every position $(x, y, z)$ within the computational domain. However, calculating the magnetic field and any other solution quantities only on a fixed discrete grid is advantageous for various reasons. It is, for example, numerically much more efficient to generate field line plots by using magnetic field values stored on a grid and use an interpolation technique than to reevaluate the Fourier sums by which the magnetic field is defined every time.

As will be discussed below, we will use a number of metrics to assess the quality of the asymptotic MHS solution in comparison with the exact MHS solution. This is also done more efficiently by using values on a grid. Finally, using a grid with a given number of grid points makes the assessment of the computational efficiency (run time) of the numerical code much easier.

Another, related point is the number of Fourier modes used in the calculation of the magnetic field and any derived quantities. For the analytical multipole boundary condition, all Fourier coefficients are in principle given analytically. Nevertheless, the Fourier series has to be truncated at a finite number of modes for computational evaluation. Usually, the truncation point has to be chosen in such a way that the Fourier series has sufficiently converged. However, because the intention of this paper is to test the numerical extrapolation method, we will actually generate artificial magnetograms from the analytical boundary conditions and use these in the same way as magnetograms based on observations. Boundary conditions based on magnetograms have a finite number of pixels and the resolution of the magnetogram determines the maximum number of Fourier modes that should be used.

As briefly mentioned above we will use standard metrics, sometimes also called “figures of merit”, (e.g. Schrijver et al. 2006; DeRosa et al. 2009) to carry out a quantitative assessment regarding the quality of the approximation of the exact magnetic field solution by the asymptotic solution. We also include a metric to compare the pressure and density in both solutions. To evaluate these metrics we assume that we have two magnetic fields, $\boldsymbol{B}_{1}$ and $\boldsymbol{B}_{2}$, which we want to compare. We assume that these magnetic fields are given on a finite three-dimensional uniform and homogeneous numerical grid with consecutively numbered grid points labelled by an index $i$. Here $1 \le i \le N$, with $N$ being the total size of our grid, i.e. $N=n_{x} \cdot n_{y} \cdot n_{z}$, where $n_{x}$, $n_{y}$, $n_{z}$ are the grid resolution in the $x$-, $y$- and $z$-directions, respectively. The metrics used are the following:

1. The vector correlation metric
  $$ C_{Vec} = \frac{\sum \limits _{i}^{N} \boldsymbol{B}_{1, i}\cdot \boldsymbol{B}_{2, i}}{\left (\sum \limits _{i}^{N} |\boldsymbol{B}_{1, i}|^{2} \sum \limits _{i}^{N} |\boldsymbol{B}_{2, i}|^{2} \right )^{1/2}} $$
  compares the local characteristics of the field vectors, where a value of 0 indicates no correlation and 1 is achieved by identical vectors.
2. The Cauchy-Schwarz metric
  $$ C_{CS} = \frac{1}{N} \sum \limits _{i}^{N} \frac{\boldsymbol{B}_{1, i} \cdot \boldsymbol{B}_{2, i}}{|\boldsymbol{B}_{1, i}| |\boldsymbol{B}_{2, i}|} $$
  measures the angle between the two fields based on the Cauchy-Schwarz inequality. The value of $C_{CS}$ ranges from −1 (antiparallel fields) to 1 (parallel fields), with 0 being assigned to perpendicular fields.
3. Two simple measures of the difference between the two vector fields are the normalised vector error metric
  $$ E_{n} = \frac{\sum \limits _{i}^{N} |\boldsymbol{B}_{1, i} - \boldsymbol{B}_{2, i}| }{ \sum \limits _{i}^{N} |\boldsymbol{B}_{1, i}| }, $$
  which is the mean error normalised by the average vector norm, and
4. the mean vector error metric
  $$ E_{m} = \frac{1}{N} \sum \limits _{i}^{N} \frac{|\boldsymbol{B}_{1, i} - \boldsymbol{B}_{2, i}|}{|\boldsymbol{B}_{1, i}|}, $$
  which is the mean error divided by the total number of grid points. The best agreement between the two magnetic fields is achieved for $E_{n} = E_{m} = 0$.
5. The magnetic energy metric
  $$ \varepsilon = \frac{\sum \limits _{i}^{N} \boldsymbol{B}_{2, i}^{2}}{\sum \limits _{i}^{N}\boldsymbol{B}_{1, i}^{2}} $$
  puts the reconstructed magnetic energy in relation to the reference magnetic energy. Identical magnetic energies lead to $\varepsilon = 1$.
6. A slightly different type of metric is the field line divergence metric, $l_{Div}$. To calculate $l_{Div}$ we trace field lines from a random point on the bottom boundary using both $\boldsymbol{B}_{1}$ and $\boldsymbol{B}_{2}$. If both field lines end again on the bottom boundary, a score $p_{i}$ is given to this point which is defined as the distance between the two endpoints divided by the length of the field line of one of the magnetic fields, say $\boldsymbol{B}_{1}$. A value can then be assigned to $l_{Div}$ by working out the fraction of the area in which $p_{i}$ is below a certain threshold. We follow Zhu and Wiegelmann (2022) who chose a threshold of 10$\% $ (0.1). Obviously, the threshold value is somewhat arbitrary, for example a value of 20$\%$ (0.2) was used by Zhu and Wiegelmann (2019). With a threshold of 10$\%$, a value of $l_{Div}=1$ implies that all tested and closed field lines end in a proximity of 10$\%$ of their length to one another.
7. To compare the pressure and density in the two solutions we use the standard linear Pearson correlation coefficients for the line-of-sight integrated (i.e. with respect to $z$) pressure ($r_{P}$) and density ($r_{D}$): a score between 0 and 1 indicates positive correlation, a score between − 1 and 0 indicates negative correlation and $r_{P} = r_{D} =0$ indicates no correlation.

For consistency with the other metrics, we will use $1-E_{n}$ and $1-E_{m}$ instead of $E_{n}$ and $E_{m}$. The best agreement between the solutions is then indicated by a value of 1 across all metrics.

## Results

Having discussed our methodology in the previous section, we will now proceed to assess whether using the asymptotic MHS solution instead of the exact solution is (a) sufficiently accurate, and (b) leads to an adequate computational speed-up of the method. We will start with the analytical multipole boundary condition to test the effect of varying the parameters $\Delta \tilde{z}$ and $a$, while keeping the values of the parameters $\tilde{z}_{0}$, $\tilde{\alpha}$ and $b$ fixed. After that we will proceed to apply the extrapolation code to the SDO/HMI magnetogram discussed in Section 3.2.2. For this case we will compare the results based on the asymptotic MHS solution and the exact MHS solution for a linear force-free case and two different MHS cases, i.e. we will vary mainly the parameter $a$.

### Analytical Multipole Case

We consider the analytical boundary condition described in Section 3.2.1 and consider seven different parameter combinations of our model, which are listed in Table 2. For all configurations we have used a normalising magnetic field strength of $B_{0} = 500$ Gauss. All calculations are carried out on a grid with the resolution $n_{x} = n_{y} = 200$ and $n_{z} = 400$. We include the first 200 modes in both the $x$- and the $y$-directions in the Fourier series. With $b=1.0$, $\tilde{\alpha} = 0.05$, as well as $\tilde{k}^{2}_{min} = (\pi /L_{x})^{2} + (\pi /L_{y})^{2} \approx 0.049348$ one obtains from Equation 15 that the maximum value of $a$ is $a_{max} \approx 0.4746697$. Based on this value, we have chosen test cases with $a = 0.22$ and $a = 0.44$. This allows us to investigate how the amplitude of the perpendicular current density affects the quality of the approximation of the exact solution by the asymptotic solution.

**Table 2:** Quantitative comparison between the exact solution by Neukirch and Wiegelmann (2019) and the asymptotic solution presented in Section 3 using the analytical multipole described in Equation 16 as boundary condition. (1) a linear force free configuration; (2 – 3) MHS configurations using a realistic value of $\Delta z = 0.1z_{0}$ together with the two different values of $a$; (4 – 5) MHS configurations using the smaller value of $a$ together with increased $\Delta z = 0.5z_{0}$ or $\Delta z = z_{0}$; (6 – 7) MHS configurations using the larger value of $a$ together with increased $\Delta z = 0.5z_{0}$ or $\Delta z = z_{0}$. We compare the vector correlation ($C_{Vec}$), the angular difference ($C_{CS}$), the complement of the normalised vector error ($1-E_{n}$), the complement of the mean vector error ($1-E_{m}$), the relative total magnetic energy ($\varepsilon $), the field line divergence ($l_{Div}$) and the Pearson correlation for plasma pressure ($r_{P}$) and density ($r_{D}$).

Case	1	2	3	4	5	6	7
*a*	0.0	0.22	0.44	0.22	0.22	0.44	0.44
$\Delta \tilde{z}/\tilde{z}_{0}$	0.1	0.1	0.1	0.5	1.0	0.5	1.0
$C_{Vec}$	1.0	1.0	1.0	0.9998	0.9993	0.9982	0.9939
$C_{CS}$	1.0	1.0	1.0	1.0	0.9999	0.9999	0.9995
$1-E_{n}$	1.0	0.9989	0.9965	0.9845	0.968	0.9552	0.9133
$1-E_{m}$	1.0	0.9996	0.9991	0.9932	0.98	0.9845	0.9568
*ε*	1.0	1.0002	1.0024	1.004	1.007	1.0346	1.061
$l_{Div}$	1.0	1.0	1.0	0.9986	0.9986	0.9993	0.9986
$r_{P}$	-	1.0	1.0	1.0	0.9999	0.9998	0.9991
$r_{D}$	-	1.0	1.0	1.0	1.0	0.9999	0.9999

We investigate the effect of varying the parameter $\Delta \tilde{z}$ by comparing extrapolation results obtained using the standard value of $\Delta \tilde{z} = 0.1 \tilde{z}_{0}$ with results for $\Delta \tilde{z} = 0.5 \tilde{z}_{0}$ and $\Delta \tilde{z} = \tilde{z}_{0}$. These choices of $\Delta \tilde{z}$ are more extreme cases, purposefully chosen to test the limitations of using the asymptotic solution. The differences between the exact solution results and the asymptotic solution results are a consequence of the widening of the domain over which the transition of the exact solution from a non-force-free state to a force-free state takes place. The asymptotic solution is independent of $\Delta \tilde{z}$ (see Equation 14 and hence remains unchanged if the value of $\Delta \tilde{z}$ changes. Additionally, for $\Delta \tilde{z} = 0.1 \tilde{z}_{0}$ we have included a linear force-free case ($a=0.0$). So overall we investigate one linear force-free and six MHS cases in total (Table 2). For each of these cases we compare the results based on the exact solution with those based on the asymptotic solution.

Table 2 summarises our results. For the linear force-free case (case 1) the magnetic fields are identical due to $f_{N+W}(\tilde{z}) = 0$ for all $\tilde{z}$. Hence, all figures of merit for the magnetic fields have a value of 1.0. A comparison of the pressure and density profiles does not make sense for this case because one would simply be comparing the stratified background model with itself.

The following two cases (cases 2 and 3) show the metrics for $\Delta \tilde{z} = 0.1 \tilde{z}_{0}$, but values of $a=0.22$ (case 2) and $a=0.44$ (case 3). For these cases, small deviations of less than $1\%$ are recorded for the two error metrics $E_{n}$ and $E_{m}$, and the magnetic energy metric $\varepsilon $. Overall, these figures indicate that the asymptotic MHS solution is an excellent approximation of the exact MHS solution for these parameter values.

For cases 4 to 7 the values of $\Delta \tilde{z}$ are increased to $\Delta \tilde{z} = 0.5 \tilde{z}_{0}$ (cases 4 and 6) and to $\Delta \tilde{z} = \tilde{z}_{0}$ (cases 5 and 7). The values for $a$ are again 0.22 (cases 4 and 5) and 0.44 (cases 6 and 7). Table 2 shows that for all these cases the error metrics $E_{n}$ and $E_{m}$ deviate most from the ideal value of 1.0. For $a=0.22$ (cases 4 and 5) they are the only figures of merit that show a larger than $1\%$ deviation from the ideal value, although as expected all figures of merit show an increase in the differences between the exact and the asymptotic solution cases with increasing $\Delta \tilde{z}$. When the value of $a$ is doubled to 0.44, all figures of merit indicate that the difference between the exact and asymptotic case is generally larger. As before these differences increase with increasing value of $\Delta \tilde{z}$. In particular the error metrics indicate more significant levels of discrepancy between the two solutions for these cases, although the values of the metrics are still surprisingly close to 1.0 even for case 7 with $\Delta \tilde{z} = \tilde{z}_{0}$, which very strongly pushes the limit for the validity of the asymptotic solution.

**Figure 5:**
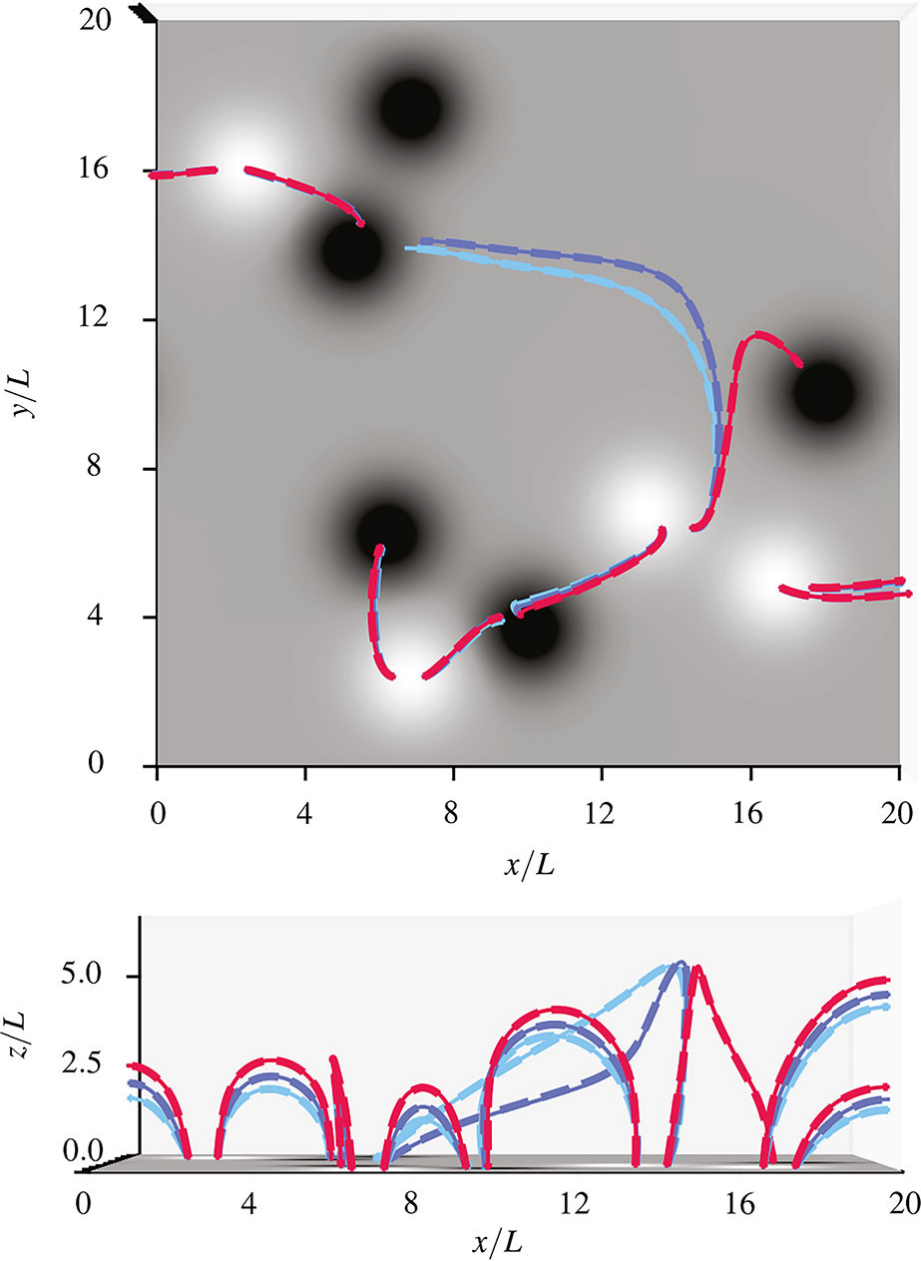
Comparison between field lines resulting from a selection of footpoints for the exact solution (dashed) and the asymptotic solution (solid) for cases 1 (light blue), 2 (dark blue), and 3 (red) projected onto the $x$-$y$-plane (top panel, top-down view) and the $x$-$z$-plane (bottom panel). If the thick dashes are centred around the thin solid lines the solutions match best, whereas a larger distance between the dashed and solid lines of the same colour indicates a larger difference between the solutions. The shape of most field lines shown is the same for all three cases. The only noticeable difference between the three cases is that of the field lines starting from a footpoint towards the right side of the positive (white) source region centred at $(\tilde{x}, \tilde{y})\approx (13.2, 6.8)$. For this footpoint the field line of linear force-free field ends at the negative (black) polarity region closest to the right hand side of the box, whereas the field lines for all MHS cases end at a negative polarity region in the top left corner. One can also notice in both panels that for this footpoint the field lines for the two MHS cases differ significantly.

Figure 5 shows a comparison between the exact and the asymptotic solution for cases 1, 2, and 3. For this comparison we display a selection of magnetic field lines starting from the same footpoints for each of the three cases. Generally, larger values of $a$ lead to an increase in steepness of the field lines below $\tilde{z}_{0}$. Therefore, for $a = 0.44$ (case 3) the magnetic field lines reach greater heights than the linear force-free field (case 1) and the MHS field for $a = 0.22$ (case 2). The footpoints of the field lines displayed have been chosen to emphasise the differences between the cases, especially in height and steepness below $\tilde{z}_{0}$. Additionally, for the field lines starting at one of the footpoints the connectivity changes between the linear force-free case and the MHS cases (see caption of Figure 5). We remark that the asymptotic solution successfully matches the connectivity of the field line of the exact solution. For this field line the largest difference between the exact and the asymptotic solution happens for case 2, as the dashed line appears to be minimally further away from the solid line than in case 3.

**Figure 6:**
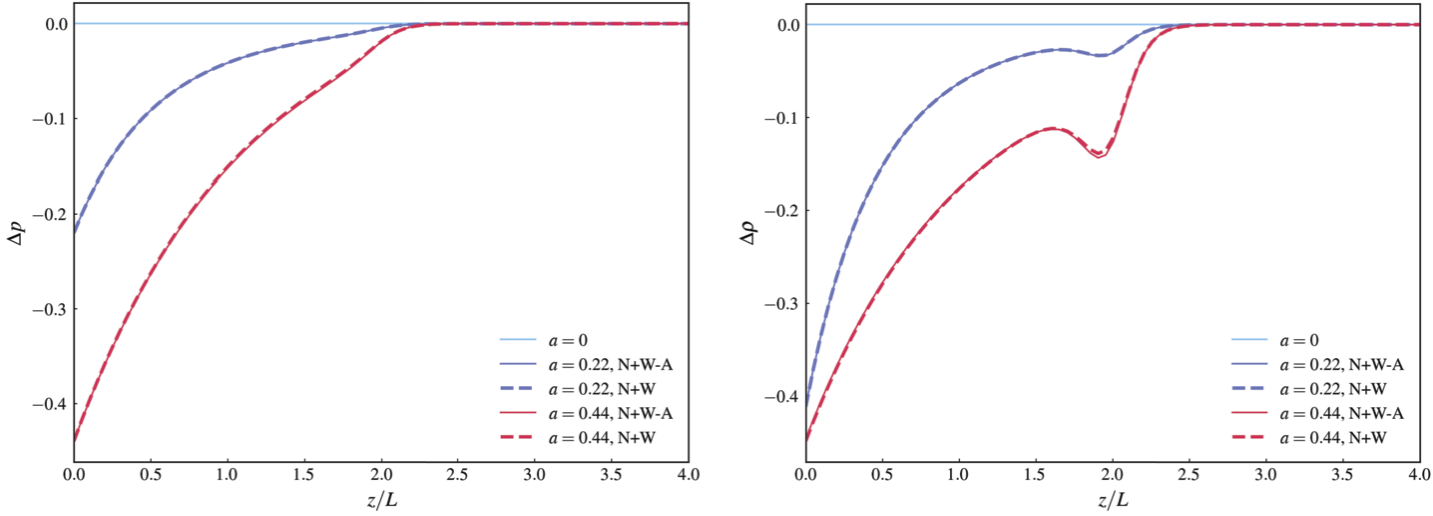
Pressure (left) and density (right) variations of cases 1 (light blue), 2 (dark blue) and 3 (red) for approximate (solid) and exact (dashed) solution at $\tilde{x}$ and $\tilde{y}$ where $B_{z}$ is maximal on the photosphere ($\tilde{z} =$ 0). $\Delta p$ is normalised by $B_{max}^{2}/\mu _{0}$ and $\Delta \rho $ normalised by $B_{max}^{2}/(\mu _{0} g_{S} L)$, where $B_{max} = \mathrm{max}(B_{z}(x,y,0))$.

Figure 6 shows a comparison between the exact (N+W) and the asymptotic (N+W-A) solutions of the variation with height ($\tilde{z}$) of $\Delta p$ and $\Delta \rho $ for cases 1, 2, and 3. The linear force-free solution (case 1) is only provided as a reference case, because by definition $\Delta p$ and $\Delta \rho $ both identically vanish for this case. To make the comparison easier we show the variation of $\Delta p$ and $\Delta \rho $ with height at fixed values of $\tilde{x}$ and $\tilde{y}$. These fixed values of $\tilde{x}$ and $\tilde{y}$ have been chosen such that for these values $|B_{z}|$ takes on its maximal value at $\tilde{z} =0$. This position has been chosen, because this is where we expect $\Delta p$ and $\Delta \rho $ to take on their largest absolute values (we note that both generally have negative values). The local minimum below $\tilde{z}_{0}$ in the density variation is caused by the term containing the derivative of $f$ in the definition of $\Delta \rho $ (see Equation 5). To avoid the negative values of $\Delta p$ and $\Delta \rho $ to cause the full pressure and density becoming negative, care has to be taken when picking the background atmosphere (see e.g. Wiegelmann et al. 2015, for an example). We also note from Figure 6 that the largest differences between the exact (N+W) and asymptotic (N+W-A) solutions can be seen around and closely to the local minimum of $\Delta p$ and $\Delta \rho $. The reason for this can be traced back to the differences between the exact and the asymptotic solutions for $\bar{\Phi}$ and its first derivative $\bar{\Phi}^{\prime}$, which are also largest around $\tilde{z}\approx \tilde{z}_{0}$, as shown in Figure 2. The slightly larger deviation seen in $\Delta \rho $ compared to $\Delta p$ is due to it having one term which depends on $\bar{\Phi}^{\prime}$, and the difference between the exact solution and the asymptotic solution is a bit larger for $\bar{\Phi}^{\prime}$ than for $\bar{\Phi}$.

The analysis above has established that for a sensible choice of parameters, a magnetic field extrapolation based on the asymptotic solution (N+W-A) approximates results based on the exact solution (N+W) very accurately. This leads to the next question: does the use of the asymptotic solution lead to savings in runtime which would justify its use in comparison with using the exact solution.

The difference in computational effort between the exact and the asymptotic solutions results from the evaluation of the function $\bar{\Phi}(\tilde{z})$ and its first derivative, $\bar{\Phi}^{\prime}(\tilde{z})$. Therefore, we test the difference in numerical efficiency between the exact and the asymptotic solution by evaluating $\bar{\Phi}(\tilde{z})$ and $\bar{\Phi}^{\prime}(\tilde{z})$ on a grid in $\tilde{z}$ of size $n_{z} = 400$. We also need to test the effect on the computing time of the dependence of $\bar{\Phi}(\tilde{z})$ on $\tilde{k}^{2}$, which we have suppressed for ease of notation. This is done by using $n_{f}=400$ Fourier modes in each of the horizontal directions ($\tilde{x}$ and $\tilde{y}$). In summary, our test grid has the size $n_{f}^{2} \cdot n_{z} = 400^{3}$. For the efficiency comparison we have used the parameter values $\tilde{z}_{0}=2.0$, $\Delta \tilde{z} = 0.1\tilde{z}_{0}$ $\alpha =0.05$, $a=0.22$ and $b=1.0$, which corresponds to case 2 in Table 2. The numerical calculations have been carried out on a MacBook Air (2020) M1 processor with 16 GB RAM.

Table 3 summarises the results of all runtime tests (for more details see the Appendix). These results indicate a significant advantage in computing time for the asymptotic solution. In this case, the calculation of the asymptotic solution was per execution an order of magnitude faster than that of the exact solution. The combined computation times of function $\bar{\Phi}$ and its derivative are 2.31 seconds for the approximate solution and 41.4 seconds for the exact solution. This implies a time advantage of $94.47\%$ over a $400^{3}$ grid and with the current implementation of the numerical code.

**Table 3:** Computation time comparison of $\bar{\Phi}$ and its first derivative for the exact solution (N+W) and the asymptotic solution (N+W-A). Shown are the average time ± standard deviation (per loop) for 10 runs (100 for-loops each), on a $400^{3}$ grid (MacBook Air M1, 2020). The numbers show that the asymptotic solution has a significant advantage in computational efficiency over the exact solution for this test case. For details of the methodology used see the main text and the Appendix.

	N+W	N+W-A
Φ̄	17.8 s ±14 ms	1.15 s ±4.76 ms
*d*Φ̄/*dz*	23.2 s ±19.5 ms	1.16 s ±1.21 ms

### SDO/HMI Magnetogram Case

In this subsection, we will present results of the very first application of our extrapolation method based on the Neukirch and Wiegelmann (2019) MHS solution family to boundary conditions based on observational data. As in Section 4.1 we will compare the extrapolation results based on the exact MHS solution with those based on the asymptotic solution. The SDO/HMI magnetogram we shall use has been presented and discussed in Section 3.2.2.

For the extrapolation we have included 239 Fourier modes in both the $x$- and the $y$-direction. In the $z$-direction our domain extends to $\tilde{z} = 20.0$. We use a grid with 222 points in the $z$-direction.

In our choice of parameter values we will be guided by the insights gained in Section 4.1. As usual for this paper we use $b=1.0$, and, as before, $\tilde{z}_{0}=2.0$. As shown in Section 4.1, a value of $\Delta \tilde{z} = 0.1 \tilde{z}_{0}$ leads to a very accurate representation of the exact solution by the asymptotic solution, and it is also in approximate agreement with the height and width of the solar transition region. We shall use a value of $\tilde{\alpha} =0.01$. We will compare extrapolation results for three different values of the parameter $a$, namely a linear force-free case with $a=0.0$, and two MHS cases with $a=0.19$ and $a=0.38$, respectively.

Table 4 summarises the results of our comparison showing that in this case the exact and the approximate model reach almost identical results in all configurations.

**Table 4:** Quantitative comparison between the exact solution and the asymptotic solution for SDO/HMI magnetogram as boundary condition. Shown are the values of the figures of merit defined in Section 3.3 for a linear force-free ($a=0.0$) and two MHS configurations. All values indicate an excellent agreement between the exact and the asymptotic solution.

*a*	$C_{Vec}$	$C_{CS}$	$1-E_{n}$	$1-E_{m}$	*ε*	$l_{Div}$	$r_{P}$	$r_{D}$
0.0	1.0	1.0	1.0	1.0	1.0	0.9994	-	-
0.19		1.0	0.9999	0.9999	1.0002	0.9994	1.0	1.0
0.38	1.0	1.0	0.9998	0.9998	1.0001	0.9991	1.0	1.0

**Figure 7:**
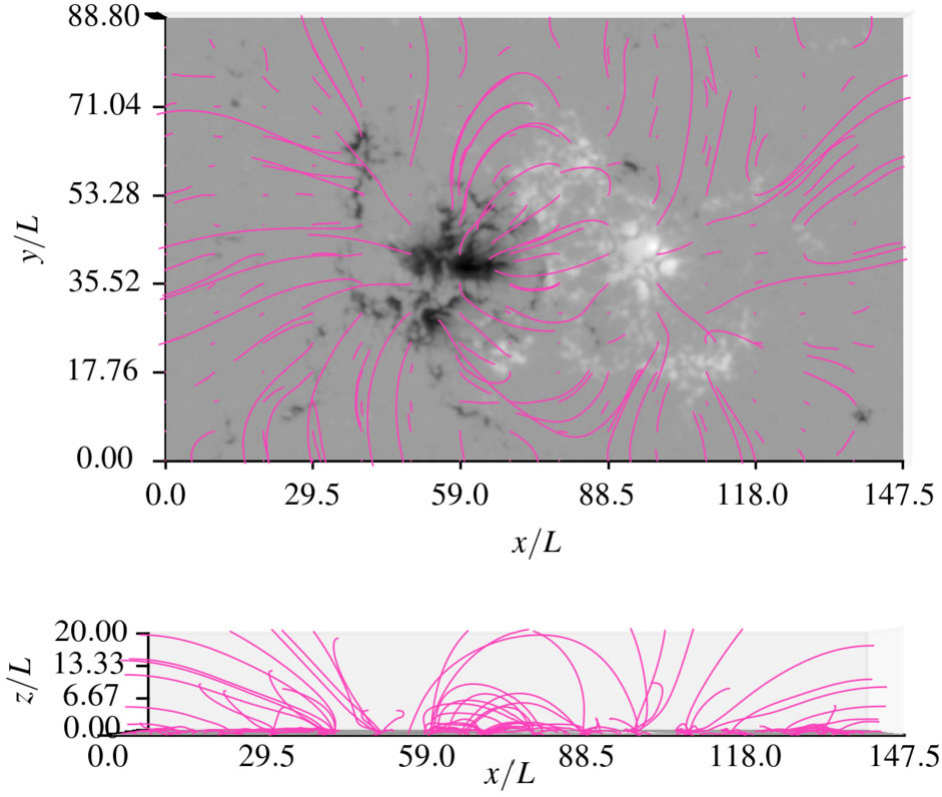
Field lines in the $x$-$y$-plane (top) and the $x$-$z$-plane (bottom) for low-a MHS configuration.

**Figure 8:**
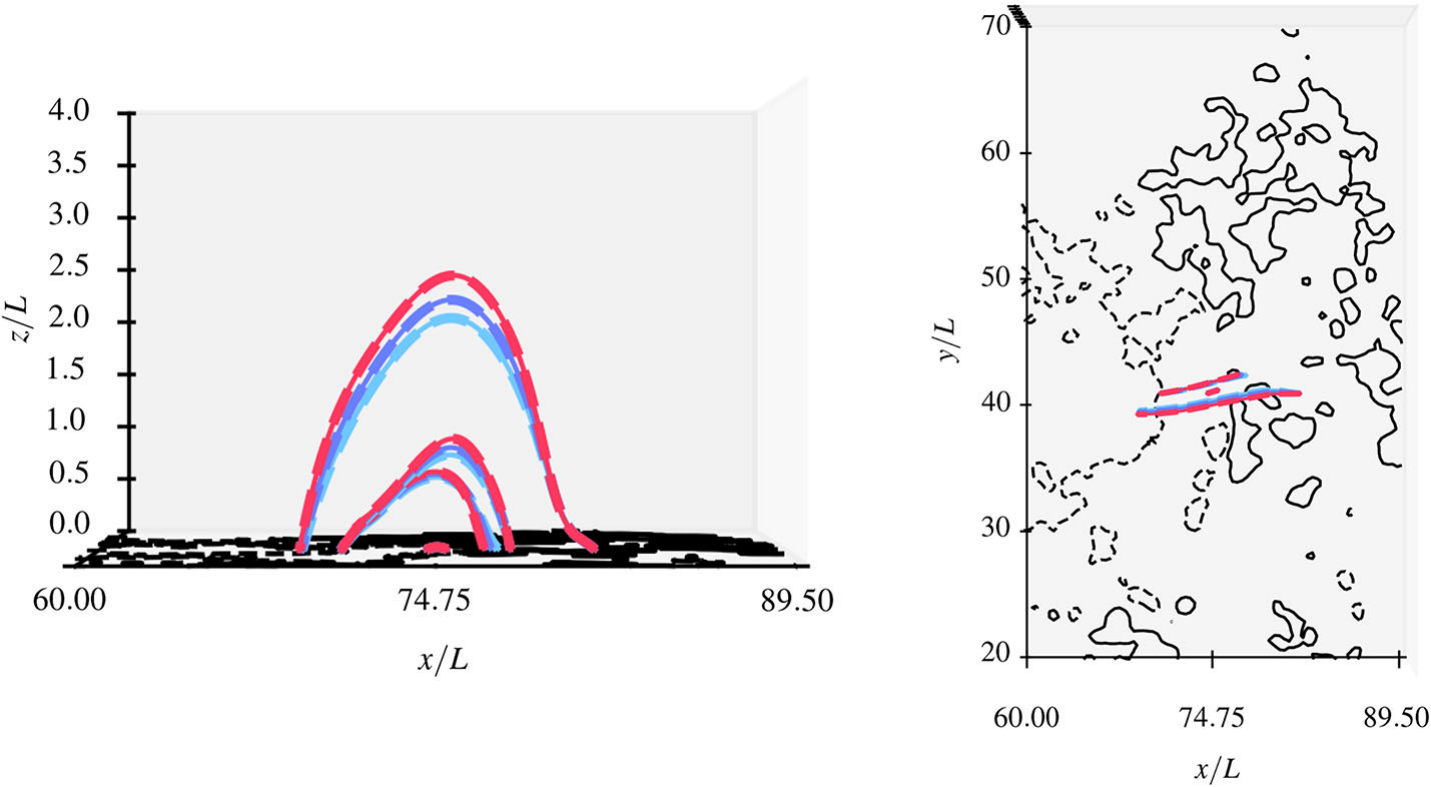
Comparison of magnetic field lines starting at four selected footpoints in the $\tilde{x}-\tilde{y}$-plane ($\tilde{z} =0$). The field lines are shown from a perspective looking in the direction of the $\tilde{y}$-axis (left panel) and looking down the $\tilde{z}$-axis (right panel). The field lines for the linear force-free case ($a=0$) are shown in light blue, for the $a=0.19$ MHS case in dark blue, and for the $a=0.39$ MHS case in red. Dashed lines represent field lines resulting from utilisation of the exact solution, solid lines for utilisation of asymptotic approximation.

For reference, Figure 7 shows the magnetic field structure obtained from the extrapolation using the asymptotic solution for the $a=0.19$ MHS configuration from a top-down perspective and a side-view perspective along the $\tilde{y}$-axis. Figure 8 shows field lines at four selected footpoints for the three different values of $a$. The differences between the field lines for different values of $a$ are most obvious in the left panel, particularly for the field lines reaching somewhat larger heights (note that $\tilde{z}_{0} = 2.0$ is the midpoint of the $\tilde{z}$-axis in the panel on the left). These differences are caused by steepening of field lines below $\tilde{z}_{0}$ with increasing $a$.

**Figure 9:**
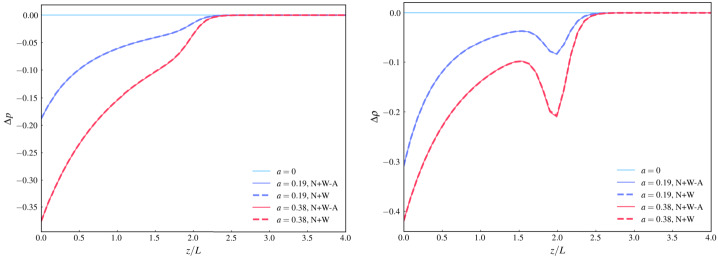
Pressure (left panel) and density (right panel) variations for the linear force-free (light blue) and two MHS configurations ($a=0.19$, dark blue), $a=0.38$, red) at $\tilde{x}$ and $\tilde{y}$ where $|B_{z}|$ is maximal on the photosphere. $\Delta p$ is normalised by $B_{max}^{2}/\mu _{0}$ and $\Delta \rho $ normalised by $B_{max}^{2}/(\mu _{0} g L)$, where $B_{max} = \mathrm{max}(|B_{z}|, z=0)$. Dashed lines correspond to the exact solution, solid lines to the asymptotic solution.

In Figure 9 we see the spatial variation of pressure and density with height $\tilde{z}$ at $\tilde{x}$ and $\tilde{y}$ where $| B_{z} |$ is maximal on the photosphere. Both pressure and density variation display behaviour that is very similar to the example based on the analytical multipole boundary conditions. Overall, all the results based on the SDO/HMI magnetogram data corroborate the conclusion that the asymptotic MHS solution is an excellent approximation of the exact MHS solution for reasonably chosen parameter values.

It remains to investigate whether the gain in computational time of using the asymptotic solution compared to the exact solution can also be found for the extrapolations based on our observational magnetogram. We have applied the same methodology that we used for the analytical multipole boundary condition. Table 5 shows the computational time needed for the exact (N+W) and the asymptotic (N+W-A) solutions for all three configurations for the SDO/HMI boundary conditions. The results clearly confirm that the asymptotic solution provides a significant runtime advantage over the exact solution also in this case.

**Table 5:** Comparison of the computation time of magnetic field vector $\textbf{B}$ and the partial derivatives of $B_{z}$ for the exact MHS solution (N+W) and the asymptotic MHS solution (N+W-A). The results show a clear computational advantage is gained by using the asymptotic solution.

*a*	N+W	N+W-A
0.0	3 m 23.0 s	38.6 s
0.19	13 m 25.8 s	1 m 26.3 s
0.38	13 m 31.3 s	3 m 39.8 s

## Summary and Conclusion

In this paper we have presented a way to significantly improve the efficiency of MHS magnetic field extrapolation based on the family of analytical MHS solutions by Neukirch and Wiegelmann (2019). These analytical MHS solutions allow for a transition between a non-force-free and a force-free domain in the direction of height above the photosphere. We have achieved this improvement in efficiency by finding a new and simple asymptotic solution which approximates the exact solution very well as long as the width of the transition zone between the non-force-free and the force-free domain is sufficiently small. The quality of the approximation of the exact solution by the asymptotic solution has been assessed by comparing extrapolation results for two different boundary conditions: one artificial magnetogram based on a period multipole structure, and an SDO/HMI magnetogram with a dominant magnetic bipole structure. For both cases, we carried out parameter studies and found using figures of merit and visual comparisons of field lines, pressure and density plots that the results based on the asymptotic solution approximate the results using the exact solution very well if the width and height of the non-force-free to force-free transitional zone are chosen to be in the same range as those of the transition region of the solar atmosphere.

After establishing that using the asymptotic solution does not lead to incorrect results, we have tested the gain in computational speed by using it instead of the exact solution. For both magnetogram case and all tested parameter combinations we found that the speed-up is significant (a factor 10 or more).

Applying our extrapolation code to the SDO/HMI data is actually the very first time that the Neukirch and Wiegelmann (2019) MHS solutions have been used for extrapolation based on observational data. We are in the process to create a Python library for magnetic field extrapolation based on the work presented in this paper, which we plan to make publicly available.

We emphasise that extrapolation methods based on analytical, linear MHS solutions such as the one by Neukirch and Wiegelmann (2019) should not be regarded as a replacement for numerical nonlinear MHS extrapolation methods. When using extrapolation methods based on analytical MHS solutions one has to bear in mind that they are restricted by the number of assumptions necessary to obtain them and by the unavoidable introduction of free parameters. On the other hand, they are computationally much more efficient and can therefore be useful as a “quick-look” tool, which is complementary to the more computationally heavy nonlinear MHS extrapolation methods.

## Data Availability

The data used in this paper are publicly available at http://jsoc.stanford.edu/.

## Appendix: Details of Computational Efficiency Comparison

We have used the IPython magic shell %timeit to time the execution time of the calculation. We have set %timeit to average the results over 10 repeats of 100 loops each. One loop in %timeit covers the computation of the function values in the whole discretised and truncated Fourier space. Each such loop in %timeit includes a for-loop over the $z$-axis of length 400, in which for each $z$, the function values are calculated simultaneously for all $k_{n}$ and $k_{m}$ through vectorisation. Hence, each loop in the for-loop (not in %timeit!) corresponds to calculating the function values for a horizontal plane $(k_{n}, k_{m})$ in the Fourier space at a specific $z_{i_{z}}$. Therefore, 10 runs of 100 loops correspond to overall 1000 calculations of the whole space per function.

The parameters $\delta $, $\gamma $, $\tilde{\delta}$ and $\tilde{\gamma}$, as seen in the definitions of exact and asymptotic versions of $\bar{\Phi}$ (Equations 10 and 13, respectively), are calculated independently beforehand for each Fourier mode. They are then passed directly to the functions whose evaluation is to be timed, such that the comparison exclusively measures the calculation time of $\bar{\Phi}$ and $\bar{\Phi}^{\prime}$ for each Fourier mode.

## Footnotes

1 See NIST Digital Library of Mathematical Functions, https://dlmf.nist.gov/15.

2 It turns out that this range can in principle be extended to allow values above $a_{max}$, but we do not investigate this case here due to the unclear mathematical complications associated with these cases.
